# Diseases of Childhood

**Published:** 1883-04

**Authors:** 


### BOOK REVIEWS.

THE DISEASES OF CHILDHOOD, WITH THERAPEUTIC INDICATIONS. By Prof. B. F. Underwood, M. D. Pp. 216. New York: A. L. Chatterton Publishing Company. 1883.

Prof. Underwood's book is a valuable one. The descriptions given of the various diseases are brief, but in the main very clear. The indications for treatment are numerous. We are glad to see prominence given to the homœopathic treatment of these diseases, rather than to the pathological theories and vagaries of the allopaths.

The worst prescription in the book is one for diphtheria, on p. 157, and reads thus: "*Calcarea chlor.*—Five to fifteen drops of Liq. calc. chlor., in one-half glass of water, given in teaspoonful doses (Dr. Neidhard)." This is a dangerous (because useless and empirical) prescription, based on the *ipse dixit* of Dr. Neidhard.

There is no specific remedy for "diphtheria," no successful "germicide." Calc. chlor., or Merc. cyan., has about as much right to such a claim as the famous "Occidental" remedy now so widely advertised as a great, never-failing cure for diphtheria.

There are a few other empirical prescriptions recommended in the book. But, on the whole, we think Dr. Underwood has given us a good book.

There are also a few typographical errors. In several places (pp. 8, 12, and 167) we see *aurum try.* for *arum tri.*, and we presume the Veratrum *viride* mentioned on p. 148 is intended for Veratrum *alb.* We do not remember ever before reading of *antimony tartar!*

